# Liver Machine Perfusion: Past, Present and Future Directions

**DOI:** 10.3390/biomedicines13112729

**Published:** 2025-11-07

**Authors:** Ivan Romic, Davor Mijatovic, Igor Petrovic, Vilena Vrbanovic Mijatovic, Goran Pavlek, Iva Martina Strajher, Vanja Silic, Karmen Jericevic, Juraj Kolak, Josip Basic, Laura Barta, Hrvoje Silovski

**Affiliations:** 1 Department of Surgery, University Hospital Centre Zagreb, 10000 Zagreb, Croatia; 2 School of Medicine Zagreb, University of Zagreb, 10000 Zagreb, Croatia; 3 Department of Anesthesiology, Renimatology and Intensive Care, University Hospital Centre Zagreb, 10000 Zagreb, Croatia; 4 Student Section at School of Medicine Zagreb, University of Zagreb, 10000 Zagreb, Croatia

**Keywords:** liver machine, perfusion, transplantation, hypothermic

## Abstract

Liver transplantation represents the only curative method for end-stage liver disorders and certain liver malignancies. Over the last three decades, advancements in immunosuppression, surgical technique, and intensive care measures have resulted in improved patient and graft survival outcomes, but a deficit of donor organs is constantly the major issue that limits our ability to reduce the mortality on the liver transplant waiting list. To address this, marginal grafts and those from donors after cardiac death are increasingly employed, but these strategies necessitated novel methods to improve the preservation and quality of liver grafts and the most promising one is liver machine perfusion (LMP). LMP evolved significantly in the past 10 years, and consequently, it is gradually establishing itself as a standard protocol at many transplant centers. However, many unresolved questions remain concerning the indications, types, and protocols associated with LMP. Therefore, continuous research is necessary to determine optimal guidelines and recommendations for its clinical application. This review aims to analyze the development of liver machine perfusion, including its different modalities underlying mechanisms of operation, and provide an overview of its historical evolution, current status, and future prospects.

## 1. Introduction

### 1.1. Historical Development of Liver Machine Perfusion (LMP)

Early concepts of ex vivo perfusion date back to the 1930s when Alexis Carrel and Charles Lindbergh first conducted organ perfusion of laboratory animals [1]. They studied mainly perfusion of the thyroid and ovaries and demonstrated that this method may preserve the viability of organs for several days. The application of these results in human organs was a difficult and slow process, so the first attempt to perfuse a liver graft was performed in the 1960s by Brettschneider et al., which showed that a liver graft may survive in canines even for 7 days after it was previously perfused ex vivo. These results were later applied to human transplants by Thomas Starzl and colleagues, who explored potential applications of ex vivo perfusion in the 1960s–1980s and demonstrated that perfusion could maintain liver function outside the body for a specific period and that it has the potential to reduce cold ischemic injury [2]. A mix of technical, logistical, and evidentiary barriers limited the further advancements of LMP and translation of it into clinical practices. In addition, static cold storage (SCS) was simpler, cheaper, easy to implement, and sufficiently effective at that time. Therefore, liver transplantation (LT) has originally relied on SCS as the standard method of organ preservation since the 1960s. However, we must highlight that Starzl’s work definitely helped establish the feasibility and rationale for perfusion-based preservation and viability assessment (VA). 

At the end of the 1980s, the University of Wisconsin (UW) solution was developed and introduced as a new SCS preservation solution. It quickly became widely adopted for organ preservation, mainly kidneys and livers, since it extended preservation times compared with older solutions. For the next three decades, SCS and UW were standard in liver graft perfusion and management during transport to the recipient center. Although SCS is simple and affordable, over time, concerns arose that this method of preservation could have negative consequences for the quality of the graft, as it offers limited protection against organ ischemia–reperfusion injury (IRI), particularly in marginal donor livers [3]. These concerns, along with advances in machine perfusion technology, better perfusates, and improved monitoring, have led to the reemergence of LMP in the 2010s. In 2010, Guarrera et al. published the first clinical series of hypothermic machine perfusion (HMP) in human liver transplantation, showing improved preservation of organs compared to SCS [4]. Another breakthrough occurred in 2013 with the world’s first liver transplant using normothermic machine perfusion [5]. Ever since, substantial improvements in technology and clinical experience have led to significant advances in LMP theory and practice. The relevance of LMP has intensified and resulted in the development of several perfusion strategies [6,7]. In the 2020s, a growing number of centers adopt LMP and ongoing trials aim to better define indications for LMP, standardize viability criteria, and optimize cost and logistics. The transition of LMP from experimental into routine clinical use was a long and progressive process over the last two decades. It was driven by advances in technology, better understanding of perfusion biology, and scientifically confirmed improvements in graft quality and clinical outcomes.

### 1.2. Why Do We Need LMP? 

The global shortage of transplantable livers and the increasing use of extended criteria donors (ECDs) and donation after circulatory death (DCD), as well as an increased demand for liver transplantation, have highlighted the limitations of SCS. Marginal grafts are more susceptible to ischemic injury, resulting in a higher rate of early graft dysfunction, as well as biliary dysfunction [8,9]. Therefore, conventional SCS does not provide optimal preservation of liver grafts, especially those from high-risk donors [9]. LMP allows dynamic preservation approaches that have demonstrated several important benefits, including improved liver function, graft survival, and reducing post-transplant complications. Consequently, LMP allows the usage of grafts that were previously discarded, potentially increasing the donor pool [9,10]. Another important benefit is that LMP, by extending preservation time, enhances the logistical and operational aspects of liver transplantation, contributing to more efficient use of healthcare resources and optimized organ transport. Thus, LMP represents arguably the most important advancement in liver transplantation over the past decade, constituting a paradigm shift in organ preservation. Simultaneously, it enables VA, and ongoing efforts aim to support therapeutic interventions and graft repair.

### 1.3. Basic Concepts and Technical Aspects of LMP

LMP refers to intracorporeal or extracorporeal perfusion of the liver with oxygenated perfusate at controlled temperatures, which aims to preserve and possibly improve the liver graft and consequently the outcomes in liver transplantation. In terms of temperature, LMP can be divided into hypothermic and normothermic protocols. While the goal of hypothermic protocols is to reduce metabolism demand and restore the function of mitochondria, sub/normothermic perfusion, on the other hand, ensures almost full metabolic activity, thus allowing real-time assessment of the function of the liver as well as delivery of therapeutic agents [6,7,10]. Fundamental studies on LMP aimed primarily to mitigate IRI, which is the main cause of liver graft damage in the perioperative process. With the development of the technique and the increasing number of studies, other pathophysiological aspects of liver damage such as cholangiopathy have also been investigated.

**Figure 1:**
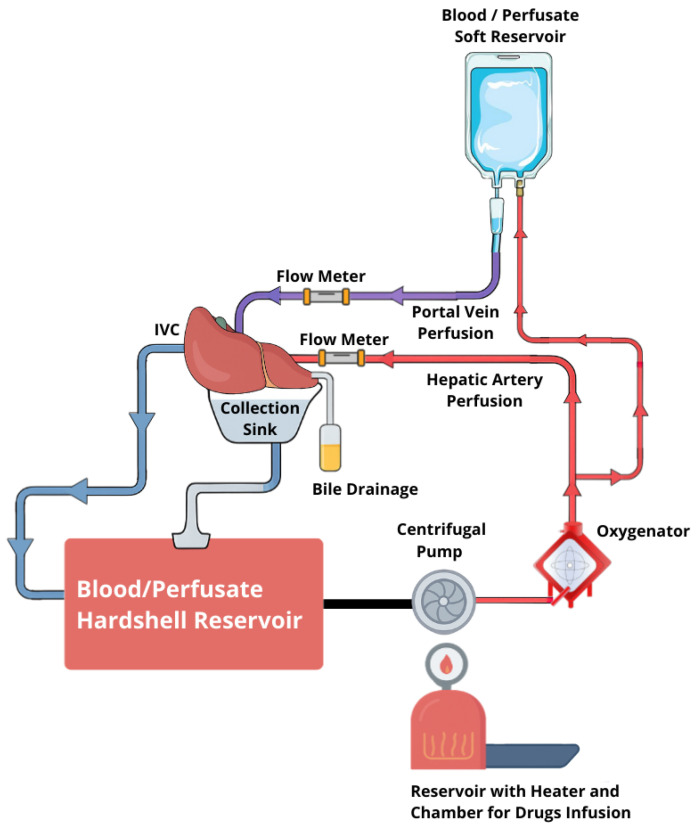
Schematic presentation of liver machine perfusion system: Sampling of perfusate and bile is typically performed at predefined time points. Recent protocols recommend baseline (0–5 min), early (1 h), key viability window (2–3 h), and extended monitoring at 4–6 h and daily during prolonged NMP. In HOPE, FMN is assessed as early as 5 min. IVC: Inferior vena cava.

Machines for liver perfusion are complex systems designed to recreate physiological conditions within the liver, but in extracorporeal settings (Figure 1). Base components include the circulatory pump system, oxygenator, temperature control units, nutrient delivery system, perfusate reservoir, and monitoring modules. LMP systems use peristaltic or centrifugal pumps to create and maintain continuous flow of the solution through liver vasculature, and the exact configuration of other components may vary depending on the type of perfusion.

Temperature is closely monitored and regulated by heat exchangers integrated into the oxygenator and perfusate reservoir. An integrated membrane oxygenator system enriches the perfusate with O_2_ while simultaneously removing the CO_2_. The perfusion fluid circulates through a closed-loop system, starting from the sterile reservoir. Modern systems are integrated with close and continuous monitoring of key parameters such as flow rates, pressures in portal vein and hepatic artery, temperature, oxygenation (pO_2_, pCO_2_), pH, glucose levels, lactates, electrolytes, and bile production. All of the data is displayed in real time and recorded. Some advanced platforms offer automated adjustment algorithms to maintain stability (e.g., pH, glucose levels) [9,10,11]. All components in contact with the perfusate are sterile and disposable. Systems include safety measures such as a flowmeter, air bubble detection, pressure alarms, and a backup power supply to maximize safety and continuity in a clinical environment.

Currently, there are seven manufacturers of liver perfusion machines, but not all of them are available on every continent. There are manufacturer-specific technical modifications, which is expected given that each company aims to demonstrate that one of its patents yields significant improvements in liver perfusion outcomes. However, the basic principles of liver perfusion are generally similar, and analyzing every single machine and its specifications was outside the scope of this article. Therefore, no specific trade names of manufacturers are mentioned herein.

#### 1.3.1. Perfusion Solutions 

Preservation and perfusion solutions are one of the key elements in the liver transplantation process. In addition to the previously mentioned UW solution, a variety of other solutions are used during organ procurement, transport, and SCS. Their roles have expanded beyond organ preservation to serve as perfusates in LMP. Moreover, these solutions are being modified to enable oxygen delivery or organ reconditioning. The UW solution, based on colloids with electrolytes, has high viscosity and is considered the gold standard for SCS of donor livers [12]. In the context of LMP, both UW and Custodiol HTK (histidine-tryptophan-ketoglutarate) are widely used in hypothermic/HOPE protocols. These perfusates may deliver oxygen during perfusion, maintain osmotic balance, and include electrolytes and medications [13]. Some studies suggest that Custodiol HTK may be optimal for HMP [14]; however, there is no clear consensus on the type of preservative fluid in LMP. Belzer MPS perfusate is another promising option for liver transplantation and LMP, particularly for marginal grafts and DCDs. It is an acellular solution containing buffers to maintain pH, osmotic agents, electrolytes, energy substrates, and antioxidants, which helps prevent cellular swelling and IRI. It is predominantly used during cold perfusion (4–10 °C) but can be adapted for normothermic conditions [15]. Steen Solution, a colloid- and electrolyte-balanced solution, was originally developed for lung preservation and ex vivo lung perfusion. While it helps maintain osmotic balance and reduce edema, it is not yet a standard or widely recognized component of liver preservation protocols, and most liver studies are conducted in animals. We found one study in the literature that used Steen Solution in humans [16]. It demonstrated that liver preservation with normothermic ex vivo perfusion using the Metra device and Steen solution is safe and yields outcomes comparable to cold storage after liver transplantation. It remains to be seen whether these results can be replicated in larger numbers and whether the positive experience from lung transplantation can be translated into LT as well. NMP is based on near-physiologic temperatures and can employ either blood-based or acellular oxygenated perfusates. Blood-based perfusates (whole blood or packed red cells with plasma) most closely approximate physiological conditions and enable reliable VA and functional testing. There is increasing use of hemoglobin-based oxygen carriers (HBOCs), such as HBOC-201, which obviate the need for red blood cells and may reduce infection risk [17]. HBOC-201-based solutions can be used across different phases of machine perfusion. Studies have suggested that livers perfused with HBOC-201 exhibit improved metabolic function and reduced liver injury markers compared with blood-based perfusates. Additionally, many centers employ device—and protocol-specific perfusates tailored to their pumps and oxygenation systems. In summary, ongoing research will continue to define optimal preservation fluids and perfusates for both transplantation and LMP. Perfusate composition can be modified in several ways and many centers employ device—and protocol-specific perfusates tailored to their pumps and oxygenation systems.

#### 1.3.2. Oxygenation 

The rationale for oxygenation during LMP is clear: timely oxygen delivery is essential to salvage ischemic tissue. The liver graft maintains metabolic activity even under hypothermic conditions; thus, oxygen delivery supports mitochondrial function, enables adenosine triphosphate (ATP) resynthesis, and mitigates IRI. Oxygenated perfusion also helps sustain endothelial integrity and microcirculatory flow [18]. From a technical aspect, in HOPE, oxygen is mixed into the perfusate or delivered via an oxygenator to the perfusate circuit at controlled partial pressures. In NMP, oxygen is delivered via an oxygenator integrated into a pump circuit and, in addition, red blood cells or plasma as perfusates provide physiologic oxygen-carrying capacity as well. Disadvantages of oxygenation include increased complexity, logistical demands, and cost, as well as safety concerns (risk of hyperoxia-induced reactive oxygen species, inflammatory responses, or air embolism). Non-oxygenated strategies are simpler and align with traditional cold storage paradigms, theoretically reducing oxidative stress. Nonetheless, there is a broad trend toward incorporating oxygenation in both HOPE and NMP protocols, supported by evidence of improved mitochondrial function, lactate clearance, energy status, and potentially better outcomes—particularly for marginal donors. Consequently, oxygenation is likely to remain a core, well-established component of LMP in the foreseeable future [19].

## 2. Types of Machine Perfusion

Several different types of LMP are developed to improve graft preservation and graft assessment prior to liver transplantation. These techniques are primarily differentiated by perfusate composition/temperature, duration, and timing of perfusion. Each type of LMP has its specific characteristics, advantages, and disadvantages as reported in Table 1. Besides hypo- and (sub)normothermic strategies, two more techniques are developed: Normothermic Regional Perfusion (NRP) and Controlled Oxygenated Rewarming (COR). In the following section, a brief overview of these protocols is provided, while the current evidence related to LMP outcomes and the comparative benefits of each protocol are discussed later in the text.

### 2.1. Hypothermic Machine Perfusion (HMP)

HMP implies perfusion of the liver with a cold perfusate, UW, or Custodiol (temperature in the range of 4–12 °C). Although it has been postulated that hypothermic machine perfusion (HMP) alone ensures a sufficient supply of dissolved oxygen to the liver graft, many authors, for the reasons mentioned above, consider that oxygenation of the perfusate is required and should constitute a standard addition to perfusion. HMP with oxygenation can be divided into two subgroups: oxygenated perfusate via the portal vein alone (HOPE) or via a dual portal vein and hepatic artery setup (d-HOPE). A pump typically perfuses blood vessel/s at low rate of flow (30–50 mL/min) and at low pressure (≤3 mmHg). The primary goal of this protocol is to restore mitochondrial metabolism and ATP production while minimizing IRI. As discussed later, evidence supports that HOPE reduces early graft dysfunction as well as biliary complications, especially in marginal donors and DCD livers [4,10,20]. A recent study from 2025 demonstrated that HMP influences the immunogenicity of donor livers in humans by modulating effector and regulatory donor-specific T-cell responses which explained decreased rejection rates in HOPE [21]. HMP is technically less complicated, less expensive, and simpler than normothermic reperfusion. It is associated a lower risk of metabolic activity, and it can offer longer storage times without the need for continuous monitoring and intervention. However, HMP represents a less physiological condition, does not allow for comprehensive real-time assessment of liver function, and does not support adequate active repair processes and therapeutic interventions.

### 2.2. Subnormothermic Machine Perfusion (SNMP)

SNMP can be considered as a bridge between HMP and NMP, and it is performed usually at around 20 °C with active oxygenation of the perfusate. Studies on animals suggest that SMP can positively impact mitochondrial function and improve graft function postoperatively [22]. Advocates of this technique suggest that a balance between a reduced metabolic demand and some degree of metabolic activity should be achieved with this temperature range (from 10 °C to 20 °C) which might be sufficient for viability testing. The main drawback is that results suggest that SNMP may maintain optimal graft conditions until around 90 min, and perfusion for more than 120 min may be counterproductive. In addition, there is a limited amount of data compared to the two other protocols.

### 2.3. Normothermic Machine Perfusion (NMP)

NMP is performed at body temperature (from 35 to 38 °C) using an oxygenated blood-based solution (red blood cell-based solutions, artificial hemoglobin solutions, or acellular solutions), and the aim is to maintain near physiological conditions [18]. Normothermic perfusion can be started as soon as the liver is out of the body (in cases of mobile machines) or it can be initiated after a certain period of SCS (SCS during transport to recipient center) or after HOPE and COR (in sequential strategies). In NMP, two independent pumps supply the portal vein and hepatic artery, respectively (150–200 mL in portal vein and 50–100 mL in hepatic artery), imitating the physiological hemodynamic. Unlike HMP, NMP allows assessment of liver, bile production, lactate clearance, glucose levels, and solution parameters in real time [23]. The addition of sorbent during NMP may reduce lactate levels and inflammatory mediators. Furthermore, other therapeutic methods such as defatting, gene silencing, or regenerative or antibiotic treatments can be administered during NPM, although most of these are still in the experimental phase. Clinical trials have shown improved graft function and reduced ischemic injury complications when using NMP, especially in DCD and steatotic grafts [24]. The main flaws in this approach include higher technical complexity, the need for blood products, shorter preservation time, and the risk of device malfunctions and other technical failures.

### 2.4. Normothermic Regional Perfusion (NRP)

**Figure 2:**
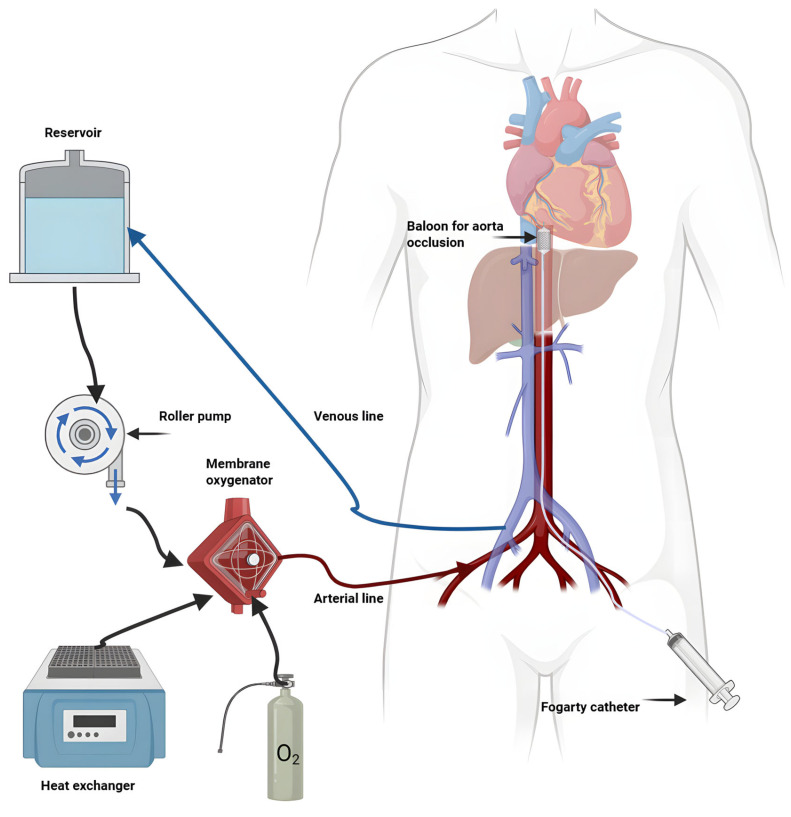
Schematic presentation of NRP.

NRP is a subtype of NMP used for in situ perfusion in a donor after circulatory death. Through extracorporeal circulation, blood flow is restored to organs in the abdomen at normal body temperature, consequently reversing warm ischemia prior to organ procurement. Abdominal NRP (A-NRP) is used for perfusion and reoxygenation of the abdominal organs while thoraco-abdominal NRP (TA-NRP) allows perfusion of both thoracic and abdominal organs. In both cases, brain circulation must be isolated. Such circulation control can be achieved by clamping supra-aortic vessels and, in TA-NRP, venting to prevent brain blood flow. The system is established in situ in the donor using an ECMO-like machine with cannulation of the aorta and a femoral vein (Figure 2). NRP is widely adopted in some countries and reports demonstrated a decreased rate of biliary complications and primary non-function in DCD liver transplants [25]. NRP requires strict protocols to avoid brain reperfusion and to maintain death determination standards. Thus, drawbacks of this procedure are logistical challenges (extra time for NRP, transport) and ethical concerns (defining death of the donor, legal complications). These limitations are discussed at the end of this paper.

### 2.5. Controlled Oxygenated Rewarming (COR)

COR represents a hybrid approach to liver reperfusion that includes incremental rewarming of the cold-stored organ with oxygenated perfusion. It is the most recent advancement in machine perfusion, and it aims to improve organ recovery and function by reducing stress during rewarming. In COR, perfusion starts at 4 °C (like in HOPE) and temperature gradually increases to normothermia (37 °C). This requires continuous temperature control and may use the same equipment as HOPE systems. Perfusate can be acellular and later switched to blood-based during rewarming, once the temperature allows. The goal is to avoid sudden thermal and oxidative stress during the reperfusion process. Initial studies report promising outcomes in older liver donors and marginal grafts. The data also suggest that COR may further help protect the liver when combined with HOPE or followed by NMP [26].

### 2.6. Is There an Ideal LMP Strategy?

**Figure 3:**
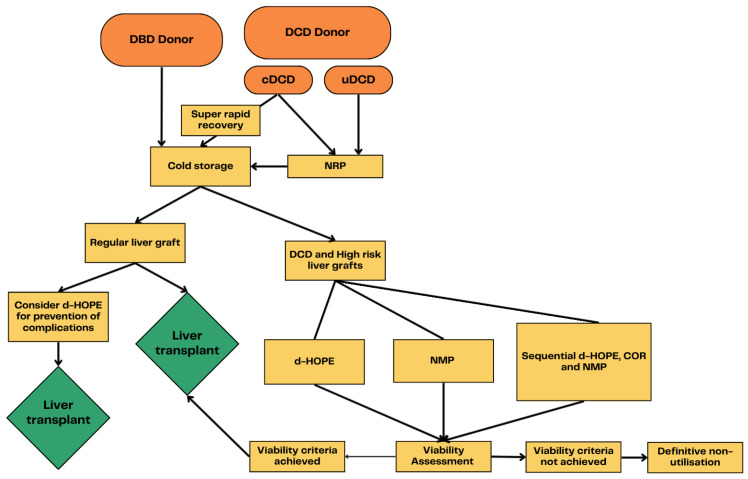
Liver machine perfusion protocols and options. DCD: donor after circulatory death; DBD: donor after brain death; NRP: Normothermic Regional Perfusion; NMP: normothermic machine perfusion; COR: Controlled Oxygenated Rewarming; HOPE: hypothermic oxygenated perfusion.

Given the availability of multiple types of liver perfusion methods, an automatic question arises: which of these methods is optimal? However, the answer is not straightforward. In the early stages of development of these machines, a certain comparative approach to evaluating different protocols existed, and this was potentially amplified by competition among manufacturers. Nevertheless, over time, following extensive experimental and clinical investigations, a paradigm shift occurred, and today, the methods are generally viewed as complementary approaches in liver perfusion, rather than strictly interchangeable alternatives. As our diagram shows, both HMP and NMP have its place in organ preservation and reconditioning (Figure 3). Moreover, some centers employ sequential strategies especially for high-risk grafts where liver reconditioning can be started with HOPE and then continued with NMP which allows adequate VA. It has been advocated that HOPE should be used even for a low-risk graft to mitigate IRI or for organizational purposes. NRP before cold preservation is unavoidable in uncontrolled DCDs and used in most controlled DCDs. There is a tendency to machine perfuse every DCD graft via HOPE, NMP, or both. COR is used mainly as a transitional stage from HOPE toward NMP in sequential strategies. Consequently, there are not many studies comparing one method to another in terms of graft improvement or survival, and majority of RCTs compared one of the methods of LMP with cold static preservation. The only study on a large number of samples that directly analyzed HOPE versus NMP is one from Wehrl et al. which concludes that HOPE-treated grafts demonstrated improved graft and recipient survival [27]. The study, however, was not RCT and only its abstract is provided by now. The best overview of HOPE vs. NMP vs. BRP comparison is provided by Patrono et al. in the current year [28]. This meta-analysis showed that HOPE and NRP, unlike NMP, were associated with a significant reduction in IC ad graft survival. However, authors highlight that studies comparing different LMP approaches are still limited and even the available literature is largely heterogeneous regarding study design, donor characteristics, and outcomes.

## 3. Current State of LMP

### 3.1. Duration of Perfusion

In addition to temperature, an important feature of LMP is the duration of the perfusion. During the early development of LMP, there was a tendency to limit perfusion to few hours in order to minimize adverse effects associated with cold ischemia and metabolic suppression. The duration of short-term perfusion is not fixed and varies by center, donor quality, and the specific purpose of perfusion (assessment vs. conditioning). Nevertheless, typical durations commonly reported are approximately 1–6 h for hypothermic machine perfusion (HMP) and up to 12 h for normothermic machine perfusion (NMP); accordingly, “short-term” generally corresponds to a period of less than 12–24 h. The development of normothermic perfusion, improved machines, better understanding of ex situ liver physiology, and advanced perfusate solutions, have led to the adoption of the concept of long-term perfusion (>24 h) [29]. The main goal of the concept was to mitigate organ shortage by perfusion machines suitable for long-term preservation. Over the past few years, substantial progress has been made in the improvement in long-term LMP. Some groups achieved NMP reaching up to 13 days so far; however, there are no reports that grafts that survived >24 h were transplanted indicating that results predominantly arise from experimental studies [30]. Ideally, long term ex situ machine perfusion of severely injured grafts unsuitable for transplantation could potentially rescue such poor-quality livers. On the one hand, this graft recovery may result from endogenous regeneration of the injured liver in normothermic, near physiological conditions. On the other side, as mentioned before, the liver can be treated with stem cells, immune modulators, antibiotics, defatting agents, or other therapeutics [30,31,32,33]. It is, however, very challenging to achieve effective long-term liver preservation. It requires a device that should mimic key components of the human body including blood flow, oxygenation, temperature, sterile environment and perfusate with components as close as human blood. A recent study from Niu et al. went one step further and evaluated the potential of long-term NMP in pediatric transplantations. They first conducted a preclinical model to demonstrate that long-term NMP of left lateral section grafts does not cause significant hyperperfusion injury. Authors conclude that this opens the opportunity to expand the use of NMP to resuscitate marginal grafts in pediatric liver transplantation [34]. An additional advantage may lie in reassessing organ viability criteria after 24–36 h, since some livers deemed non-viable at the early assessment (after 4–5 h) may subsequently prove viable. While recent developments of long-term NMP are promising, further research and translation into clinical practice is necessary to optimize long-term perfusion protocols.

### 3.2. Viability Assessment

The advent LMP has enabled pre-transplant VA of liver grafts. Given the potentially deleterious and, in many cases, fatal consequences of graft dysfunction, the importance of reliable VA is evident. VA, however, remains a significant challenge, largely because there is no universally accepted definition of viability. Functional assessment of the liver can be divided into hepatocellular (evaluated using lactate clearance, perfusate pH, transaminases, or bile production) and cholangiocellular viability which is assessed by the specific biochemical composition of bile. Several criteria of viability emerged by combining parameters such as the Cambridge, Groningen or Birmingham criteria. Currently, none of these are dominant in clinical practice, but continuous efforts are made to standardize VA in future.

For a long time, it was assumed that VA is only possible during NMP. Typically, perfusion parameters (vascular flows, resistive indices), perfusate analytes (pH, lactate, transaminases, glucose), and bile analytes (pH, glucose, bicarbonate) can be used to assess deceased donor livers [35]. Unfortunately, NMP parameter thresholds are still not adequately established, but the most commonly reported ones are presented in Table 2. Given the impracticality of VA parameter assessment during HMP, efforts have focused on identifying other quick and reliable predictors of graft injury that can be measured during this type of LMP. For a long time, VA in HOPE was based on perfusate lactate, transaminases, and LDH measurement. Consensus guidelines from the European Liver and Intestine Transplant Association (ELITA) pointed out that clinical evidence supporting the use of biomarkers for VA in HMP is scarce, but it also suggested that the predictive potential of flavin mononucleotide (FMN) presents a major breakthrough in the field [36]. A study from Eden et al. presented international validation of various mitochondrial injury biomarkers measured during HOPE before LT which serve as predictors of graft injury [37]. The study included 473 perfusates and it demonstrated that mitochondria-derived FMN values in perfusate are predictive for graft loss, cholangiopathy, and kidney failure after liver transplantation. These are promising results that show that it is possible to compensate for the main drawback of HMP relative to NMP, which is the inability to assess graft function under physiological conditions. Another study from 2025 aimed to validate the predictive potential specifically of FMN and it showed that early assessment of FMN enables the identification of grafts at risk for EAD [38]. A recent study from Wehrle et al. support routine utilization of FMN during NMP, not just HOPE [39]. Their single-center result showed that it reduced complications and improved graft survival. At the same time, it reduced transplant costs due to reduced post-transplant complications. Novel markers such as microRNAs, ATP production, or proteomic profiles offer the potential to improve graft evaluation; however, the practical use of these biomarkers is limited by the lengthy processing times required, which might delay transplantation decisions.

In conclusion, in recent years, various parameters have been proposed to determine graft viability during NMP and even in HMP. However, a definitive, reliable, and widely accepted indicator for confirming a liver's suitability for LT has yet to be established. This lack of standardization in graft quality assessment impedes the reliability of scientific results related to LMP. An optimal approach may be combining perfusion parameters (both hepatocellular and cholangiocellular viability criteria) with biomarkers.

### 3.3. Rationale for LMP and Current Evidence 

Ischemia–reperfusion injury is a major determinant of early graft dysfunction after liver transplantation. Therefore, many studies on LMP have focused on the potential of machine perfusion to mitigate IRI. Preclinical studies consistently showed improved mitochondrial function, higher adenosine triphosphate/adenosine diphosphate ratios, and reduced oxidative stress after LMP, which led to conclusion that LMP is superior to cold preservation in protecting against liver IRI [40,41]. Hypothermic oxygenated perfusion (HOPE) and subnormothermic protocols reduce inflammatory response, preserve sinusoidal endothelium, and decrease markers of cell death in animal models [42,43].

The rising prevalence of donors with fatty liver has driven the use of LMP not only as a preservation tool, but also as a platform to assess and potentially repair marginal and steatotic grafts. Experimental studies and studies on discarded grafts suggest that prolonged NMP can enable safe multi-day organ preservation, continuous functional monitoring, and in some cases, metabolic interventions such as defatting protocols [44,45]. Long-term machine perfusion of fatty livers has recently demonstrated the technical ability to work for >7–12 days, maintaining hepatocellular and biliary function, with automated perfusate composition and nutritional supplementation. Two key paradigms when managing steatotic grafts currently coexist: (1) NMP as a therapeutic option to reduce the lipid content and improving marginal grafts, (2) NMP primarily as a diagnostic and assessment tool to decide whether to accept or reject the organ. Promising clinical results were published in 2023 by Petrono et al. (on 10 cases) which presented successful transplantations of steatotic grafts after NMP and VA [46]. Cirelli et al. presented two patients who received severely steatotic donor livers following resuscitation and VA using DHOPE-COR-NMP [47]. Although both steatotic grafts functioned well during NMP, the clinical outcomes in both patients were complicated. Therefore, authors suggested that LMP alone is not the definitive answer to risks that pose steatotic liver grafts and transplantation of severely steatotic liver grafts still remains a risky challenge.

Considering the lack of clinical studies, we agree that consistent clinical benefit of NMP as a defatting method has yet to be proven. There are currently no randomized investigations on this topic, but one RCT was officially registered last year, and we await those results with great interest [48].

### 3.4. Cholangiopathy Prevention

Post-transplant cholangiopathy remains a major leader of morbidity and graft loss. Non-anastomotic strictures (NASs) are diffuse, intra/extrahepatic strictures remote from the anastomosis, usually linked to peribiliary gland/plexus injury and ischemia–reperfusion injury and should be distinguished from anastomotic strictures from broader “ischemic-type biliary lesions” constructs used variably across the studies. Lack of uniform definition and outcome reporting complicates cross-trial comparisons and pooled estimates. Evidence across HMP modalities demonstrated that HOPE/D-HOPE resulted in significant reduction in biliary complications, including NAS, versus SCS in DCD grafts, supporting it as a preventative measure for ischemic cholangiopathy [10].

NMP landmark clinical trials showed less hepatocellular injury, but effects on NAS/ITBL are heterogeneous and often underpowered as biliary endpoints were secondary and unclearly defined. Recent synthesis of the studies showed no uniform NAS reduction across NMP studies, though centers report favorable cholangiocellular markers (e.g., bile secretion, bile pH) during perfusion [49]. Studies on NRP suggest lower rates of ischemic cholangiopathy/NAS compared to rapid recovery, likely via immediate reperfusion and mitigation of warm ischemia before procurement [50]. Regarding COR and hybrid protocols, early studies are promising mechanistically, but there is still insufficient data on NAS prevention for clinical claims [51]. In conclusion, among LMP strategies, HOPE and NRP currently have the clearest clinical signals for reducing ischemic biliary cholangiopathy/NAS in DCD context. However, for reliable final evaluation, a standardized NAS classification and prespecified biliary endpoints in future studies are required.

### 3.5. Improved Graft Function 

The next step in translation form basic studies to clinical practice was proving that positive initial results on molecular, cellular, and histological levels including mitigation of IRI also translate into improved graft function and, ultimately, improved graft survival and better long-term transplantation outcomes. To evaluate this, a reliable methodology for graft assessment was required. There are two possible approaches to assess liver graft treated with LMP. First, it can be performed during LMP by measuring specific viability parameters, or it can be indirectly assessed by analyzing clinical parameters or histopathological parameters after the transplantation (for example EAD or graft survival).

After substantial advances in LMP over the past decade and its firm integration into clinical practice, most prominent authors from the field of LMP have published in 2022 an important, comprehensive review of current knowledge and recommendations related to LMP and suggested future objectives [52]. All relevant randomized and non-randomized studies for different types of LMP published by 2022 were collected and results confirmed safety and efficiency of LMP in assessing organ viability, optimizing organ quality, and extending preservation time. Special focus was given to the prevention of IRI, the role of oxygenation, and the need for nomenclature standardization and standardization of VA. In addition, authors recommended a new combined (sequential) approach: HOPE, followed by long-term normothermic perfusion which could be an optimal strategy for marginal grafts. It was later incorporated into ELITA guidelines as mentioned below. One year later, in 2023, a study from Schlegel et al. provided all current strategies that improve the quality of donor livers and discussed the potential of bioengineering techniques to design optimized grafts during LMP [53]. Apart from known LMP benefits in terms of organ preservation and reconditioning, authors highlight the huge potential of long-term normothermic liver perfusion associated with interventions that could mitigate IRI. Novel strategies to improve the quality of risky donor livers such as cell-based therapies, genetic modulation, and functional liver scaffolds were also presented. In the same year, the first meta-analysis on LMP was conducted by Parente et al. and it included seven RCTs on NMP or HOPE protocols [12]. The results showed that both perfusion techniques were found to reduce overall biliary complications and non-anastomotic strictures and were associated with significantly lower rates of early allograft dysfunction (EAD). Outcomes, however, were limited to a 1-year follow-up after liver transplantation so authors highlighted the need for comparative RCTs and large real-world cohort studies in the future. The scientific community obviously addressed this need and from that study, until now, more than 10 meta-analyses of LMP were published. This is a sign that there is a positive trend towards conducting high quality RCTs. The majority of RCTs compared HOPE to SCS in terms of early graft dysfunction and postoperative complications and these report improved early graft function with LMP versus SCS. Almost all landmark studies have demonstrated that LMP, both NRP and ex situ LMP, confers benefits for DCDs and marginal liver allografts (in comparison with standard-criteria organs), including reductions in complications such as EAD and ischemic cholangiopathy [54,55,56,57,58,59,60,61,62,63,64,65,66,67].

The OCS Liver PROTECT Randomized Clinical Trial showed in 300 patients that NMP reduces both post-transplant EAD and ischemic biliary complications [67]. Other trials showed a 50% lower level of graft injury in NMP, a lower risk of non-anastomotic biliary strictures in HOPE, and increased utilization of ECD livers in NMP [10,68,69].

Wiesel et al. showed in a nationwide analysis on more than 7000 DCD donors that the use of LMP in the United States increased organ utilization and improved DCD graft survival in LT. The patient survival was equivalent in DBDs and DCDs. These impressive clinical results definitely justify the use of LMP in practically all DCD donors [70].

In 2024, Garzali et al. conducted a systemic review and meta-analysis of studies comparing LMP and SCS in terms of EAD [71]. Eight studies, published from 2019 to 2023, were included. The conclusion was clear: LMP in the preservation of liver grafts showed a significant reduction in EAD and retransplantation compared to SCS. Such favorable clinical results represent an important step in a wide implementation of LMP in clinical practice. Recent metanalyses from 2025 confirmed results from Garzali with regard to mitigating EAD [72,73,74]. Viana et al. demonstrated similar results for NMP and, in addition, showed that NMP is associated with lower non-anastomotic biliary stricture rates [75]. The increasing trend of RTCs and seven meta-analyses that are published already in 2025 is a sign that LMP remains a hot topic in realm of LT.

## 4. Current Guidelines and Recommendations

A broad implementation of LMP in clinical practice necessitates addressing several critical questions: What is the optimal perfusion temperature? When should we perfuse? How long should we perfuse? What is the optimal perfusion pressure and flow? What is the optimal oxygen carrier? What are good criteria for viability assessment? What drugs should be administered during perfusion?

Generally, there is a lack of clear guidelines on LMP; nevertheless, as over the past two decades, the application LMP has moved from the realm of clinical exploration to routine clinical practice, several relevant recommendations have been published recently. Using the modified Delphi method that involved a panel of experts, in 2025, ELITA (the European Liver and Intestine Transplant Association) published guidelines regarding selection, approach, and criteria for DCD liver graft assessment [36]. More than 700 articles were included for full-text analysis. Authors highlight that this guidance is provided in a rapidly evolving clinical landscape and advances in the field of LMP in the coming years will undoubtedly prompt new refinement of the strategies; however, some important conclusions were given. First, the panel recommended changes in terminology and classifications. For example, the term “discarded” should be avoided, and the term “non-utilization” should be preferred. Regarding VA, panelists agree that the best-described and studied parameters are biliary pH, bicarbonate, and glucose levels measured in relation to perfusate values. Guidelines indicate that graft steatosis and predicted rates of primary non-function, EAD, acute kidney injury, complication-free survival, overall biliary complications, ischemic-type biliary lesions, 3-month and 1-year graft, and patient survival should be considered to define a graft as not suitable for transplantation. In situ abdominal NRP alone or combined with ex situ perfusion methods can be used to recondition and assess controlled DCD livers for subsequent transplantation.

In addition, a stepwise approach to the management and assessment of livers arising from DCDs was given. It recommends that some type of LMP should be considered in all cases of both DBD and DCD donors. For livers deemed suitable for transplantation, an ex situ LMP should be considered in order to prevent IRI. For livers deemed unsuitable for transplant, two strategies are described. First, after cold preservation, only NMP with VA can be employed. Second, initial perfusion can be performed using HOPE and then COR can be performed prior to NMP and VA. The further decisions on graft transplantability depend on VA. The future will show us if clinicians are ready to use LMP for every single liver as some centers do, but most experts form the panel agree that ex situ NMP is recommended to assess DBD livers that would not be transplanted otherwise. At the end, centers are encouraged to follow ELITA 2025 assessment pathways and local ethical frameworks, prioritizing graft assessment over unproven therapeutic ambitions.

The American Society of Transplantation currently recommends the use of LMP in DCD donors, steatotic grafts, and older-age donors, where traditional preservation is associated with a risk for the graft viability. Some centers advocate for HOPE or D-HOPE as routine procedures in DCD donors, given its low complexity and proven decrease in biliary complications [76].

Highlights from the 2024 ILTS (The International Liver Transplantation Society) Congress warrant discussion as well [77]. The congress convened a multidisciplinary, global community of experts, with LMP emerging as a central theme. The transformative potential of LMP in liver transplantation, particularly for donation after circulatory death (DCD) grafts, was emphasized. HOPE has reinforced its role as an established preservation technique in liver transplantation, advancing to IDEAL-D Stage 4 level of evidence. NMP continues to demonstrate value in functional graft assessments. Participants suggested that substantial benefits may arise from innovations in graft regeneration, spontaneous defatting during extended perfusion, and immunologic and molecular modifications. NRP attracted increasing attention for its capability to optimize the utilization of older DCD grafts, with promising results emerging from AI-driven in situ viability predictions. 

In conclusion, recommendations emphasize a tailored approach, integrating risk factors of the donor, perfusion technique, as well as expertise of the transplantation team. Further large-scale trials are required to establish standardized clinical pathways and guidelines to ensure safe and effective implementation of LMP.

## 5. Discussion 

A notable shift from reliance on experimental toward real-world evidence and cost-effectiveness analyses reflects growing confidence in the safety and efficacy of LMP. Machine perfusion technologies have demonstrated great results in optimizing graft quality, mitigating IRI, and enhancing the use of marginal and DCD liver grafts. There is sufficient evidence that LMP reduces EAD and biliary complications, and enhances these types of grafts [4,6,7,10,20,49,55,57,58,59,60,61,62,63,64,65,66,67,78]. Nevertheless, we need to be clear and objective; as of now, there is no definitive evidence that LMP universally prolongs patient or graft survival compared to cold static storage when regular liver grafts are used [56]. Improved viability of marginal grafts contributes to better short-term outcomes, but more large-scale studies are needed to see if this may translate into improved long-term survival.

We may conclude that all techniques of LMP for the liver have the potential to mitigate IRI and prolong the preservation period, but, at same time, they offer different benefits and are associated with various limitations. Thus, there is currently no robust evidence to support the superiority of one technique over others. Nevertheless, as previously mentioned, it appears that all methods will coexist, with each potential approach offering benefits in particular clinical circumstances.

Many efforts are given to put the idea of repairing injured donor livers during LMP into practice as it might further increase the utilization of ECD livers. Application of defatting agents is currently explored in clinical trials, whereas other therapeutics require further research or optimization before entering clinical research.

While some authors advocate for the routine use of machine perfusion in liver transplantation, we prefer to maintain a skeptical attitude. First, if LMP replaces the standard SCS preservation completely, it will significantly increase the cost of liver transplantation; however, the procedure might be unnecessary, particularly for healthy liver grafts from young donors or grafts with short cold ischemia period. Second, we are still not aware of potential adverse effects of LMP on the liver, having in mind potential technical failures, graft damage during preparation, or machine errors. And third, as the method is relatively novel, there are still uncertain long-term benefits. We must take into account the interests of machine manufacturers, who will advocate for the widespread use of LMP in all or most transplants. Thus, we unequivocally endorse the application of LMP in liver transplantation, albeit with restricted indications, ideally for DCD and ECD grafts. For these grafts, and those from very old donors (>70) a sequential approach by combining HOPE, COR, and NMP might be an optimal strategy that prevents rapid temperature changes [79].

Concerning research, it is difficult to conduct reliable comparative studies, as randomization can be challenging due to ethical considerations and logistical constraints. In addition, there is a wide spectrum of liver and donor conditions, and it is difficult to assess liver damage in the peri-transplant period, particularly the effect of the primary disease and intensive care measures on liver tissue. These challenges should be addressed with robust methodologies and global collaborative trials so that LMP research can yield more meaningful and applicable results.

The limitations of different LMP protocols are briefly outlined in Table 1. Bearing in mind the complexity of liver physiology and the numerous functions that the liver has, it is clear that machine creation is a complex process. Evidence-based benefits exist for high-risk grafts, especially for DCDs; however, superiority over SCS for healthy grafts with short cold ischemia time has not been clearly demonstrated. Despite substantial efforts and recent ELITA guidelines, variable standardization (heterogeneity in protocols and viability criteria) hampers comparability. Understandably, long-term outcomes are still missing since the method is still relatively novel.

Besides the aforementioned limitations of LMP with regard to graft function, there are some practical barriers to LMP use. First, the cost of the machine results in a high initial capital expenditure. This disadvantage is more pronounced when we add compatible consumables, disposable kits, cannulas, and backup systems. The LMP program requires sophisticated staff education and financial compensation for perfusionists and surgeons. Both surgeons and nurses/technicians should be familiar with the perfusion process, and continuous support from LMP engineers and manufacturers is a requisite. Organizational issues and device availability should be discussed as well. Incorporating LMP into existing transplantation processes, from procurement through transport, and transplantation logistics is challenging, and a lot of effort is required to successfully implement it. At the end, device-related risks such as perfusion circuit failure, contamination, and air embolism risks should not be ignored. LMP should definitely be implemented in high-volume centers that have expertise in liver transplantation, the necessary infrastructure, and stable finances. Implementation of NRP is highly heterogeneous worldwide (Table 3), shaped by legal definitions of death, ethical frameworks, and transplant policies. Unlike device-based ex situ protocols, NRP involves in situ extracorporeal circulation in the DCDs, raising regulatory and ethical barriers which remain debated and vary across jurisdictions. In some countries (e.g., USA, Germany, Switzerland), the reestablishment of regional blood flow is prohibited by law after circulatory death, as it is considered incompatible with the definition of death. These legal and ethical aspects of transplantation required thorough elaboration by leading professional societies in the field of liver transplantation. ESOT provided a consensus document with evidence-based recommendations on the use of NRP in DCDs [80]. They recommended the use of A-NRP in uDCD procedures for 1–4 h, in preference to ISP and static cold storage when ethical, technical, and logistical requirements are met; however, solid organ grafts from uDCD NRP donors need to be used with caution, weighing the risk of patients’ continued waiting against the risk of adverse graft outcome. Also, they suggested minimal logistic requirements for NRP and technical characteristics of this method and discussed ethical considerations of this method. Authors stressed the importance of further research on the necessity of ex situ organ perfusion following NRP. Previously mentioned ELITA guidelines encompassed recommendations related to NRP as well. In that document, it is recommended that NRP can be used to recondition and assess cDCD livers for subsequent transplantation. In addition, NRP should be used to recondition and assess uDCD livers, though additional ex situ LMP preservation should be considered in these grafts prior to transplantation. VA should be based on flow rates, perfusate lactates, and transaminases, but livers can be evaluated for up to 2 h for cDCD and up to 4 h for uDCD.

The American College of Physicians (ACP) published a statement of concern regarding NRP in 2021 [81]. The paper outlines that NRP-cDCD raises profound ethical questions regarding the dead donor rule, beneficence, and justice. They consider that NRP-cDCD appears to violate one of the ethical foundations of organ donation, the “dead donor rule”, which specifies that donors cannot be made dead in order to obtain their organs and that organ retrieval cannot cause death. This “rule” requires that cessation of circulatory and respiratory function is irreversible, and authors of ACP statement consider NRP to serve as a way to re-initiate circulation which means that the patient is, in fact, successfully resuscitated. ACP recommends the use of NRP-cDCD be paused since these concerns have not been adequately considered to date. The paper notes a policy–practice gap, with low case volumes and widespread unregulated adoption, and calls for further professional and public discussion on NRP-cDCD with the aim to provide national standards to ensure safety, consistency, and preservation of public trust in organ donation.

In contrast, some societies advocate the use of NRP in all DCDs considering that it mitigates the detrimental impact of warm ischemic time. The British Transplantation Society supports the routine use of NRP, and recommended that it should be used in all DCD retrievals where there is a trained team competent to perform it.

The Organ Donation and Transplantation Alliance provided an overview of risks and benefits of NRP, but concluded that NRP can improve graft and patient outcomes in multiple organs, increase the number of potential organs for transplant, and has significant cost savings in comparison to ex vivo perfusion machines. In 2024, the American Society of Transplant Surgeons provided a paper describing NRP standards [82]. The workgroup, comprising experts in NRP, DCD, and transplantation, formulated recommendations regarding NRP which encompass a range of considerations, including preprocedural communication, procedural guidelines for NRP teams, uniform terminology to clarify the NRP process, and standards for mentorship and credentialing of NRP practitioners.

At the end, The American Society of Transplantation (AST) declared their position on NRP in 2022 [83]. They endorse innovation in modalities such as NRP which can increase organ use, reduce organ injury, and improve recipient outcomes. Based on the current procedural, ethical, and legal assessments on NRP in DCD donors, the AST supports the use of this technique and the development of associated strategies that promote its broader clinical implementation. But they also highlight the critical ethical analysis and the necessity of legal clarifications.

In summary, although substantial scientific debate is ongoing and further clinical research on NRP is anticipated, most relevant societies support the implementation of NRP, for which there is sufficient evidence that it improves transplantation outcomes from DCD donors and enables reliable viability assessment. Therefore, NRP represents a highly disruptive technology with a transformative impact on DCD transplantation.

**Figure 4:**
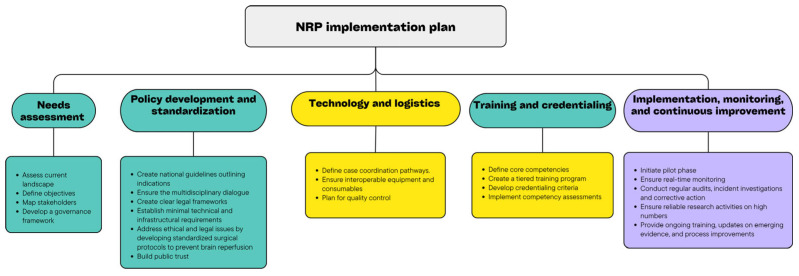
NRP implementation plan.

All emphasize, however, that there are challenges related to the moral and legal aspects of the procedure itself. Therefore, ongoing open scientific discussions, the introduction of clear legal frameworks, standardization of the procedure, and familiarization of both the expert community and the broader public with these issues are essential. Analyzing the previously mentioned guidelines, we have prepared a schematic outline of a plan for implementing NRP into national transplantation programs (Figure 4). Such structured policy-mapping frameworks could guide centers or countries planning to introduce NRP to their clinical practice. The process involves several key segments that need to be addressed.

Implementation should begin with needs assessment meaning that centers should evaluate their current DCD liver transplantation outcomes to identify specific unmet needs. Step two includes engagement of relevant societal and professional groups, not just medical staff, but also ethicists, legal professionals, religious representatives, and patient advocates, ensuring the multidisciplinary dialog ensures transparency as well as builds public trust and unites professional, ethical, and societal perspectives. The third key segment is regulatory and policy alignment; national transplantation laws, definitions of death, and institutional ethical guidelines should be reviewed and aligned. Finally, proper education and communication are of paramount importance. Structured training programs should be provided for transplant surgeons, anesthesiologists, and coordination teams covering technical procedures, donor management, and data documentation. A clear communication plan regarding NRP for donor families and donor hospital personnel should be established. Legal clarification either through updates to the Uniform Determination of Death Act (UDDA) or other legal advisories should ensure adherence to the principles of the dead donor rule. It is the optimal way to preserve public trust and remove any perceived misalignment or legal barriers to NRP donation either as a clinical or a research protocol. Once the NRP program starts, continuous evaluation and improvement should be a priority. Pilot NRP programs should start under ethical supervision with predefined clinical endpoints (graft survival, biliary complications, and organ utilization). Together, these steps create a policy implementation map that bridges technical feasibility and real-world implementation.

## 6. Future Directions

We witness ongoing refinements of perfusion machines and protocols, accompanied by an increasing number of manufacturers. Moreover, an expanding cohort of liver transplant centers is adopting LMP, suggesting a positive trends toward LMP adoption. The advent of long-term organ perfusion offers substantial potential to enhance organ evaluation and selection, to recondition or repair marginal grafts, and, ultimately, to expand the graft pool available for transplantation.

From a technological perspective, the primary focus is on developing portable devices capable of automated perfusion control to ensure optimal conditions during transport. Significant efforts are also directed toward enabling long-term organ preservation, extending to several days or even weeks [84]. A lot is expected from increased preservation times which will allow for additional research in terms of repair and regeneration. 

Other technical advances are related to perfusate formulations, monitoring sensors, and simplifying design. Recent development of artificial intelligence (AI) technologies, combined with real-time monitoring algorithms, shows a promising future for optimizing automated machine liver perfusion, enabling optimal perfusate flow rate and pressure for maximum organ repair and longer life outside the body.

The most significant next step would be the ability of LMP not just to preserve but also to repair or rejuvenate the liver, and research into administering therapeutic agents during perfusion is required to achieve this goal. Several interventions, such as defatting protocols, gene silencing, antioxidant strategies, and immunomodulation, are being explored, aiming to improve graft quality and viability, especially in high-risk organs. The concept of cryopreservation has also been explored; however, current studies are limited to animal models, and the potential future clinical application remains unclear.

Identifying reliable biomarkers for assessing the graft viability during the perfusion process would allow better recipient selection and prediction of potential complications. Standardizing the criteria of viability (such as bile production, lactate clearance, and hemodynamic stability) is crucial for more precise and consistent decision-making and broader use of LMP in clinical practice.

Finally, wider implementation of LMP in clinical practice will depend on cost-effectiveness, training of the transplantation team members, and crucially, further development of international consensus guidelines. It should also be emphasized that NMP offers distinct logistical advantages. By extending preservation time and enabling daytime scheduling of liver transplantation, NMP can reduce night-time procedures, improve staffing efficiency, and decrease fatigue-related surgical risks [53]. These operational benefits, repeatedly highlighted by Schlegel et al., provide an important counterbalance to the higher upfront costs of implementing NMP programs [85].

At the end, a proper definition of graft risk and personalized medicine would tailor perfusion protocols based on specific donor and recipient characteristics, which could improve outcomes and further expand donor eligibility.

## 7. Conclusions

Liver machine perfusion is a dynamic and one of the hottest topics in the field of liver transplantation. It shifts preservation from static cooling to active organ management. Trends suggest that LMP will play an increasingly critical role in the future of organ transplantation, particularly in high-risk liver grafts where it demonstrated great promise in optimizing graft quality and mitigating ischemia–reperfusion injury. The final goal of LMP is improving both efficiency and success rates while increasing the number of available livers. Further innovation, supported by studies on a larger sample and universal guidelines, will be key to wider implementation of LMP. 

## Figures and Tables

**Table 1:** Comparative overview of liver perfusion techniques.

	HMP/HOPE	NMP	NRP	COR
General concept	Hypothermic, oxygenated perfusion that focuses on mitochondrial protection	Ex vivo normothermic perfusion maintaining near-physiologic metabolism for assessment/conditioning	In situ regional perfusion at normothermia, often pre-transplant, aims to extend viability in situ	Transitional, controlled rewarming with oxygenation between HOPE and NMP
Temperature	4–12 °C	35–38 °C	35–37 °C	12–35 °C
Viability criteria	Transaminases, LDH or lactate levels; FMN.	Functional metabolism and hemodynamics.	Functional viability indicators in situ.	Early metabolic indicators at subnormothermia.
Advantages	Mitochondrial protection; reduced IRI; simpler logistics	Direct functional assessment; potential for conditioning and repair during long-term NMP	In situ viability assessment and extended preservation	Smoother transition; preserves oxygen delivery during warming
Disadvantages	Limited assessment of full metabolic function; limited interventions during perfusion	Resource-intensive; more complex to operate; risk of inflammatory response; longer perfusion times; requires blood products	Invasive/logistical complexity; ethical questions; in situ exposure may have limits on conditioning	Limited standardization; interpretation of intermediate markers is evolving
Future perspectives	Better standardization; development of reliable biomarkers; technological integration	Therapeutic conditioning; automation and AI	In situ functional conditioning; expanded adoption	Better standardization of sequential protocols; early-phase markers development

HMP: hypothermic machine perfusion; HOPE: hypothermic oxygenated perfusion; NMP: normothermic machine perfusion; NRP: Normothermic Regional Perfusion; COR: Controlled Oxygenated Rewarming; IRI: ischemia–reperfusion injury; FMN: flavin mononucleotide.

**Table 2:** Current thresholds for viability assessment.

Marker/Parameter	Perfusion Type	Common Threshold/Time Window	Clinical Relevance
Lactate clearance	NMP	<2.5 mmol/L, within 2–3 h	Hepatocellular metabolic activity
Bile volume	NMP	>10 mL in 2–4 h	Billiar viability
Bile pH	NMP	>7.4 during perfusion	Cholangiocellular integrity
Bile bicarbonate	NMP	>18–20 mmol/L	Cholangiocyte transport activity
Bile glucose	NMP	Lower than perfusate glucose	Intact biliary epithelium
Hemodynamic stability	NMP/HMP	Stable flows with physiological pressure	Vascular integrity
FMN release	HOPE	Early rise within 5 min is a negative predictor	Mitochondrial injury, graft loss risk

**Table 3:** Current status of NRP in different countries/regions.

Region/Country	Status of NRP	Notes
Spain, Italy, UK	Routine, national protocol	Prospective multicenter evidence; part of standard DCD practice
France, Belgium, Netherlands	Permitted, variable adoption	Defined within DCD framework, but regional uptake differs
Austria, Denmark, Sweden	Pilot/limited	Small single-center experiences
Germany, Switzerland	Not routinely used but there is ongoing discussion	Legal/ethical constraints; definition of death precludes in situ reperfusion
East Europe, Asia, South America	Unclear, but evolving in some countries	No clear regulatory basis; exploratory phases in most centers
USA	Adopted and piloted at multiple centers, but case volume is small	No single national standard governing NRP; ongoing debate on ethical and legal issues
Greece, Slovakia, Bulgaria, Slovenia	No documented NRP cases in public sources	Implementation of NRP depends on evolution of DCD programs
Croatia	No DCD program by now No LMP implementation	Initial phase of needs assessment; multidisciplinary dialogs recently started

## Data Availability

No new data were created or analyzed in this study.

## Abbreviations

The following abbreviations are used in this manuscript:

LMP	Liver machine perfusion
SCS	Static cold storage
UW	University of Wisconsin
IRI	Ischemia–reperfusion injury
HMP	Hypothermic machine perfusion
SNMP	Subnormothermic machine perfusion
NMP	Normothermic machine perfusion
IVC	Inferior vena cava
ECD	Extended criteria donors
NRP	Normothermic Regional Perfusion
COR	Controlled Oxygenated Rewarming
HOPE	Hypothermic oxygenated machine perfusion
D-HOPE	Dual hypothermic oxygenated machine perfusion
